# Proofs of the Health-preserving Properties of the Jussieua Grandiflora

**Published:** 1840-06

**Authors:** Samuel A. Cartwright

**Affiliations:** Natchez, Miss.


Art. III.—*Proofs of the health-preserving properties of the Jussieua Grandiflora or Floating Plant.* By Samuel A. Cartwright, M. D. of Natchez, Miss.

To a considerable number of plants all ages have ascribed therapeutic properties, or such as cure disease. I propose to introduce to the notice of the profession a plant possessing hygienic properties, or such as prevent disease.

The region of country where I found this plant growing in the greatest abundance is in Lower Louisiana, the most southern portion of the territory of the United States, except the peninsula of East Florida. It lies between the 29th and 30th degree north latitude, and between the 80th and 92d degree of west longitude. New Iberia, Donaldsonville, and New Orleans are situated near the northern boundary of this region, and the Gulf of Mexico constitutes its southern limits. With due allowances for the large lakes, bays and indentations of the Gulf of Mexico, it contains about seven thousand square miles—a territory about equal in extent to the State of New Jersey. Not more, however, than one-third is arable land; the other two-thirds being subject to annual inundation from the Mississippi river, and composed of cypress and palmetto swamps, small lakes, bayous and *Prairies Tremblant* or shaking prairies. The tillable land is exceedingly fertile and is but a few feet above high water mark. There is nothing like a hill in this whole region of country with the exception of a few Indian mounds. The soil is entirely alluvion of recent formation. The high or arable land lies on the margin of the lakes and bayous. It is not, generally, more than from half a mile to a mile in width; and gradually slopes back from the bluff or high banks of the lakes and bayous with a gentle declivity of three or four feet to the mile to a cypress swamp, beyond which is a palmetto swamp, then comes a *prairie tremblant*, in the centre of which is commonly a small lake. Beyond the *prairie tremblant* is another palmetto swamp, then a cypress swamp, then comes high land again, extending from the swamp with a gentle ascent to another bayou or lake. The bayous are very numerous; some of the principal ones are the Lafourche, the Blue, Terre-bonne, Cailliou, the Black, Buffalo, Alligator, Boeuf, Teche, Atchaffalaya, &c. Barataria, Palourd, and Tampelier are the largest lakes. Berwicks, Atchaffalaya and Vermilion are the most important bays. The water in all the lakes and bayous, except Lafourche, is entirely stagnant and has no motion but what it receives from the winds, except very near the Gulf of Mexico, where a slight motion is given to it by the tides. Even when the Mississippi river inundates the low lands and swamps, there is little or no current in the water which finds its way out of the banks of that river. The bayou Lafourche is nothing but a natural canal or aqueduct leading from the Mississippi river to the Gulf of Mexico by a shorter route. It is a small embra sure of the Mississippi river, and is only navigated by barges and light steam boats. It differs from these bayous, properly so called, which are nothing more than stagnant canals or natural channels not generally wider than the Schuylkill, extending from the south in nearly parallel lines to the Gulf, and remarkably straight in their course; or connecting the different lakes with one another.

The first quarter or half quarter of a mile on the right and left of the bayous, in the uncultivated parts, is generally covered with a magnificent growth of live oak, *Quercus virens.* The branches of this tree spread out horizontally to the distance of thirty feet or more from its trunk. The balance of the high land, extending to the cypress swamp, is covered with a lofty forest rendered almost impenetrable in its natural state by reason of its dense undergrowth of cane. The cane is from ten to twenty feet in height. The culms of the cane are not generally more than an inch or an inch and a half in diameter. The culms stand so close together that a bird can with difficulty fly through them, and an active man can scarcely travel half a mile an hour in the thicket.

The principal trees which overshadow these cane-brakes are remarkably large and tall. The long gray moss, the *Tillandsia Usneoides*, several feet in length, suspends itself in thick clusters from their boughs. The wild grape vine, which is often near a foot in diameter, ascends their trunks and spreads itself among their branches; and the ligneous rope, *the liandre*, often out-climbs the grape vine, and having tied the branches of the trees together, descends again to the earth, takes root, climbs another tree or twines its smooth and polished surface around the parent stem. Besides the live oak and several other species of the *genus quercus*, the principal trees of the arable, or high land, are the sweet gum, *Liquidambar Styraciflua*, the blue and white ash, *Fraxinus Quadrangulata*, and *F. Acuminata*, hack-berry, *Celtis Crassifolia*, the Pacane tree, *Juglans olivaeformis*, which bears the nut called pacanes, or pecons; the honey locust, *Gleditschia Triacanthos;* the cotton-wood tree, *Populus Argentea*, a very different tree from the *Populus Canadensis,* also called cotton-wood tree, of more northern latitudes. The cotton-wood tree of the south, though a tall tree, two or three feet in diameter, differs, I believe, from all other trees, in having roots like the grasses—the roots being fibrous, not racemose, as other trees, but consisting almost entirely of long slender fibres, or radicles, without a caudex. The sassafras of the genus laurus, grows in this district of country to a large size, its trunk being often two or three feet in diameter. The sumach belonging to the rhus genus, although a dwarf in size, is deserving of notice, as it is a certain indication that the land on which it is found growing is not subject to inundation.

The cypress swamps in rear of the arable or high land, have no undergrowth of cane—but are thickly beset with *cypress knees,* rising from two to six feet in height, erroneously supposed by Michaux, to be excrescences of the root of the cypress tree, the *Cupressus Disticha.* They differ as much, however, from the root of the cypress tree, as the misletoe differs from the oak on which it is found growing. They are evidently parasites of the root, entirely different, either from the cypress tree or its roots, being double hollow cones closed at both ends, one apex descending several feet perpendicularly into the earth, and the other as many above the earth's surface. They are constituted of a dense alburnum, covered with bark, having no branches, either above or below, and no connection with any other substance, except the radicles of the cypress root, to which they owe their origin. The swamps in which these singular vegetable productions grow, are principally shaded by cypress trees, the lords of a southern forest, which afford a most durable and valuable timber. The palmetto swamps lie almost posterior to the cypress swamps, and extend to the margin of the prairie-tremblants. These swamps do not contain any large trees, except the wild olive, a tree which grows in very wet places, has an ash-colored bark, smoother than the cypress, and bears a berry about half the size of the olive, and very like it. Its leaves fall off soon and turn red before falling; but the principal growth of the low swamps is the palmetto, or dwarf, fan palm, *Chamærops Humilis*, rising from five to ten feet in height, having a long fusiform root. It would seem that the *flabellum*, or fan-like expansion, which constitutes the external portion of this shrub, exists originally in miniature in the root. Because if the top of the root be cut off in the spring, it will nevertheless send its flabelliform leaves, not with acuminate points, as in the natural state, but premorse, as if they had been clipped off with a pair of shears. When land containing a growth of palmetto, is reclaimed by ditches, the practice pursued by some of the planters is to thrust an iron crow-bar, perpendicularly into each palmetto root, before putting the land in cultivation, otherwise the root, which is too deep to be extirpated by the plough or the hoe, will continue to send up premorse leaves which will be very troublesome. It is somewhat singular, that the palmetto land, when reclaimed from inundation, is the very best land in the world for cotton. The cotton plant, on palmetto land does not grow tall, but is short-jointed and contains more bowls or pods by one-third, than the cotton plant on cane land.

The lakes and bayous of lower Louisiana abound with fish and contain great numbers of alligators ; also alligator-gars, a fish without scales, partaking of the nature of an alligator and a cat-fish. Though much smaller, they are far more dangerous to persons who venture into the water than the alligator. Nearly the whole surface of many of the bayous and a considerable surface of many of the lakes, in all that part of Louisiana below 30° of latitude, are covered, in a greater or less degree, with the Jussieua Grandiflora, the plant, which possesses hygienic or health-preserving properties. Besides the Jussieua Grandiflora, I observed a considerable number of other aquatic plants, both phænogamous as well as cryptogamous. Among the aquatic plants were the *Callitriche aquatica* or water star grass; the *Lemna minor* or Dutch meat; the *Riccia natans* or floating liverwort; the *Nympha cærulia* with its broad leaves; the *Isnardia palustris* with its grass like leaves; besides the *Rafflesia arnoldia*, the *Lemnochæris Humboltii*, the *Hydrocotyle vulgaris*, and a few others. On the bays, the fucus natans or gulf weed, was very common. Nearly all the aquatic plants, with the exception of the Jussieua grandiflora, the Lemna Minor, and the Fucus Natans, had more or less attachments to the soil by means of their roots. The fucus natans was only found in salt water. The lemna minor is a very small and insignificant plant. The Jussieua grandiflora, however, is excluslvely aquatic. It is a large flowering plant, which grows three or four feet above the surface of the water, and gives the water on which it grows the fallacious appearance of a natural meadow. The root is several feet in length, is jointed, about half an inch in diameter; lies horizontally on the water, but an inch or two below its surface. Each joint sends up the culm or stem of the plant, and around each joint of the root; at the foot of the stem are a great number of radicles or hair-like roots, some of which float on the surface of the water, and others dip down towards the bottom or fasten themselves to old logs. These radicles or little roots, often have adhering to them an inky kind of paste or substance, which they collect from the water, and no doubt constitutes the nourishment or proper aliment of the plant to which they belong. The roots, radicles and radicle leaves of the Jussieua Grandiflora, form such a dense covering to the water, as to constitute a bridge sufficiently strong to enable snakes and grass-hoppers to cross over the stagnant pools in which it grows. I travelled forty miles in a canoe through bayous and lakes, which were almost entirely covered by the Jussieua Grandiflora and intermixed with a number of other aquatic plants. I was often unable to see any water at all, except in the track made by the canoe. Although very frail, and easily pushed aside or broken, this floating plant afforded considerable resistance to the progress of the canoe. On the wide lakes and bays, the winds often detach large masses of this and other aquatic plants, which being driven about by the waves, and one detachment forced upon another, constitute what are termed *floating Islands*—which are often strong enough to bear the weight of a man in a recumbent posture. The Jussieua Grandiflora, together with the other aquatic plants mentioned, are not only found on the lakes, bays and bayous, but they constitute the sub-stratum of that singular and non-descript species of savannah called the *prarie tremblant.* These prairies are constituted in the first instance of a vast assemblage of aquatic plants. On this vegetable stratum, intermixed with the debris of other vegetable substances, a number of grasses and terrestrial plants like parasites, fasten themselves and grow. The whole is formed into a complete vegetable mattress, strong enough to support a man in a crawling position, but not sufficiently firm to enable him to walk upright. It is also too firm to admit of the passage of a boat or canoe. When the foot is placed upon it, the whole mass trembles; hence the French name *prairie tremblant*, and the English name *shaking prairie.* It is said that if a hole is cut in it, fish may be caught with a hook and line.

The facts on which I rest the hygienic or health-preserving properties of the Jussieua Grandiflora, are—

1st. That it purifies all stagnant water in which it grows:

2. The remarkable exemption of the inhabitants of that section of Louisiana from malarious or miasmatic diseases.

1. The water on which the Jussieua Grandiflora grows differs essentially from other water, similarly circumstanced, where this plant does not grow. Although I visited the country in which the plant is indigenous, during a very dry and hot season, in the month of June, I found the stagnant water of the lakes and bayous, inhabited by this plant, as pure to the sight, taste and smell as if it had just fallen from the clouds. Near the Gulf of Mexico, however, the water of the bayous was impregnatəd with salt. The water also of Bayou Black, although fresh, had a darkish appearance—owing to a chemical affinity between some ferruginous matter in the soil, and the oak trees and leaves which had fallen into the water. The water of Bayou Black, although of a dark color, was free from any disagreeable taste or smell. It contained no green scum, and was considered to be equally good and palatable as cistern water; except near the Gulf, where the water is impregnated with salt; the inhabitants, who reside on the margins of the stagnant lakes and bayous of that part of Louisiana, drink no other kind of water.

I could discover no other cause for the remarkable purity of the stagnant water in the lagoons, swamps, lakes and bayous of lower Louisiana, than the aquatic plant under consideration.

North of the region where the Jussieua Grandiflora flourishes, there is the same kind of alluvial soil, formed by depositions of the identical rivers, which form the soil of Lower

Louisiana, yet stagnant water in hot weather, becomes exceedingly impure, beyond the limits in which the plant under consideration is found. The soil, therefore, cannot occasion the purity of the water of Lower Louisiana, because the same kind of soil, a little further north, has not the same effect. Nor can the purity of the water be owing to the salt or sea water; because the water is equally pure, wherever the aquatic plant grows, whether in salt water or fresh.

I think it may be fairly inferred, therefore, that the aquatic plant known by botanists under the name of Jussieua Grandiflora, consumes or feeds upon those substances, which in other situations corrupt and vitiate stagnant waters in a warm climate.

2. The remarkable health and longevity of the inhabitants, and their exemption from malarious and miasmatic diseases. The fact that the region of country in which the aquatic plant abounds, is exceedingly healthy, can be established beyond cavil or dispute. It nevertheless contains more stagnant water and swamps, than any other inhabited district of the same extent in the United States.

The country immediately north of the line bounding the growth of the floating plant, which is about the thirtieth degree of north latitude, like that south of thirty, is alluvial, contains lakes, swamps and stagnant waters—is covered with nearly the same vegetable productions; but its atmosphere is evidently insalubrious, its stagnant waters impure, its inhabitants sickly, and human life of short duration; while the country of the aquatic plant, immediately south of it, contains a wholesome atmosphere, pure water, healthy, and long-lived inhabitants. It may be supposed that this country is too new and too thinly inhabited to form any correct estimation of the health and longevity of its inhabitants. Such a supposition is erroneous. Although a considerable part of the region abounding in the aquatic plant is uncultivated and almost uninhabited, yet a very considerable portion of this territory has been settled nearly a century. A large colony from Nova Scotia emigrated to it before the revolutionary war. Some of the settlements south of New Orleans contain more free white inhabitants to the square mile than the oldest and most populous settlement in Pennsylvania.

It may be said that the inhabitants are the descendants of French and Spanish, and consequently no just comparison can be drawn between them and the descendants of the English. It is true that a large portion of the inhabitants are of French extraction. A large settlement of them on the Lafourche, within this region, were born north of the United States, in the cold latitude of Canada. Colonel Sparks, an intelligent sugar planter, who resides on the Bayou Lafourche, in the midst of the colony which emigrated from Nova Scotia more than half a century ago, informed me, in 1831, that a great number of the emigrants were still living. He took me to a number of their houses, and his statements were confirmed by the inhabitants themselves. I saw more than a sufficient number of gray heads and healthy looking children to remove all skepticism in reference to the health and longevity of the inhabitants. Besides the French population, this particular section of country has spread through it a number of Italians, Spanish, Dutch, German, Irish, English and Scotch. It also contains emigrants from almost every State in the Union. The negro population is also considerable, and is remarkably healthy and long-lived. It contains more negroes over one hundred years of age, than five New England States put together, including the total population, white and black. The population of this land of aquatic plants owes its origin to so

4

many different nations, that it is not uncommon for the Creoles or natives of the country, even when uneducated, to speak with great ease three or four different languages. If it were true, which it is not, that the French people are exempt from miasmatic diseases, such as bilious, remittent and intermittent fevers, it would prove nothing; because the Germans, Spanish, Italians, Scotch, Irish and English, together with the negroes and emigrants from the States north of Louisiana, are all in this land of aquatic plants singularly exempt from such diseases. But neither the French nor any other race of people are thus exempted when they cross the line which terminates the growth of the floating plant. It is therefore a fair inference that this plant, by consuming the impurities of the stagnant waters, prevents the generation of miasmata, and thus acts as a prophylactic against bilious fevers and othermiasmatic diseases.

I am aware that the inhabitants of the country themselves attribute their peculiar healthfulness to the influence of sea-breezes. Out of the region of the floating plant, sea-breezes, however refreshing and beneficial to some constitutions, have not been found to exert any prophylactic power in preventing miasmatic diseases. It is not probable that sea-breezes would do more good for the sea coast of Louisiana than for the sea coast of Georgia, Carolina, Virginia and Maryland.

In the summer of 1831, I travelled extensively through Lower Louisiana, and am fully convinced from what I saw and heard that the particular district of country in which the floating plant abounds is pre-eminently healthy, while those sections of the State, similarly situated but where the aquatic plant was not found, are grievously afflicted with malarious diseases.

I visited, among others, the plantation of M. Rochelle, on a small bayou, near Berwick's bay. The dwelling houses stood on the high ground, about a quarter of a mile from the bayou. The space between the bayou and the houses was occupied by a swamp through which a canal had been cut to afford access to the high ground or bluff, on which the dwellings stood. M. Rochelle, a few years previously, had the trees, covering the swamp, in front of the houses, cut down, in order to gain a better view of the bayou, and obtain a freer circulation of air. As I passed up the canal or ditch, through the swamp, I perceived, on each side, the decaying timber lying in the water, which was entirely stagnant. In many places the water was not sufficient to cover the ground. On ascending the bluff and looking around, I ascertained, that besides a swamp of a quarter of a mile in width and three miles in length in front of the plantation, there was also an immense swamp in the rear, running back to a *prairie tremblant* ; and on the lower side of the plantation was another bayou of stagnant water, and on the upper side a thick forest and cane break. I thought, at the time, that, if the country contained a sickly spot, this was the one.

The Jussieua Grandiflora, however, grew in profusion in all the waters around, whether these waters were in the bayous, or in the swamps ; and whether they had communication with the bayous or were isolated stagnant pools, they were found to be pure and transparent—free from any offensive taste or smell.

M. Rochelle had fifty-three negroes living on this plantation and his white family consisted of about a dozen persons. He informed me that himself, and all the family, white and black, except the younger children, were natives of Rockingham county, Virginia—that he had resided on his plantation with this large family, nine years, during which time no deaths had occurred, either among the whites or blacks, young or old—that there had been not more than three or four cases of sickness during any year—that these cases were slight and required little or no medical treatment. His neighbors confirmed this statement, and gave nearly as good an account of themselves. The negroes with whom I met, all looked healthy, happy and contented.

The next evening I put up at a house containing about twenty white persons and no negroes. The patriarch of the family was a Kentuckian by birth. He married a Spanish woman, who, dying, had left him a widower with several children. He afterwards married a French widow, with two or three children, whose former husband was a German. The children by the last marriage as well as by the former marriages, together with a few aunts and relatives, swelled the whole family to about twenty. No less than four languages, English, Spanish, German and French, were spoken by the same family, living under the same roof.

I got two of the sons of the old gentleman by his first wife to take me in a canoe up Bayou Black. They were with me several days, and, as they spoke four languages, were of great use in enabling me to collect information respecting this country inhabited by the floating plant and polyglot people.

If I have been misinformed in reference to the health of this section of country, then also are great numbers, in and about this city, deceived in this respect equally with myself. Numbers of people in and about Natchez have visited this region of country; some have removed to it; a few have been living in it for years. All, with whom I have conversed, concur in the same opinion of its healthfulness. It is true, they differ in regard to the *causes* of its singular salubrity; some ascribing it to the proximity of the sea and the sea breezes; others to the large open prairies on its western border, in and near that part of it called Attakapas, without recollecting that the inhabitants of Terrebonne and Lafourche, who reside very remotely from these large prairies and secluded from them by intervening forests, are equally if not more healthy than those living near them.

Having ascertained the facts of the purity of the stagnant waters, and the health of the inhabitants in this section of country, I examined the subject attentively on the principles of that new and interesting science, called, by the French, Political Arithmetic.

The Parishes on and below the thirtieth degree of north latitude are the following: Ascension, Parish of Orleans, (not the city of New Orleans,) St. John the Baptist, St. James, St. Martins on the line of 30°; and the following below 30° and south of New Orleans, viz: St. Mary, La Fayette, St. Charles, St. Bernard, Jefferson, Plaquemine, Assumption, Lafourche, and Terrebonne. These Parishes in 1830, not including any part of the city and suburbs of New Orleans, contained a total population of 83,943—27,778 white persons and 46,165 negroes. They contained thirty-eight persons over one hundred years of age, whereas the whole of the New England States, with a population 1,954,704, including upwards of 20,000 negroes, only contained thirty-five persons over one hundred years of age. France with thirty-six millions of inhabitants only contained five hundred and thirty-seven persons over one hundred years of age. If France were as favorable to longevity, as the land of the Jussieua Grandiflora, it ought to contain upwards of sixteen thousand individuals over one hundred years of age.

By instituting a comparison on the data afforded by the census of 1830, it will be found that, in all that part of Louisiana where the Jussieua Grandiflora grows, for every hundred white males and females between twenty and thirty years of age, there are upwards of one hundred children under five years of age; whereas, in that part beyond the region of the Jussieua Grandiflora, the number of children to every hundred white males and females, between twenty and thirty years of age, is only *eighty-six*, being more than fourteen per cent. less than in that part of Louisiana inhabited by the floating plant.

Again, for every one hundred males and females in those parishes of Louisiana inhabited by the floating plant, there are seventy-nine children between five and ten years of age; but beyond the region of the floating plant, the number is only fifty-seven.

The proportional number in Upper Louisiana of those between ten and fifteen, and fifteen and twenty, is from eight to ten per cent. less than in that part of Louisiana inhabited by the Jussieua Grandiflora.

Taking the number of persons between twenty and thirty years of age in both districts as the standard of comparison, the number of citizens between thirty and forty years of age is eight per cent. greater in the district of the floating plant than in the district north of the region inhabited by it.

The number of white persons between forty and fifty, between fifty and sixty, and between sixty and seventy is from three to four per cent. greater in that part of Louisiana inhabited by the Jussieua than in the district lying north of it. The surest proof of the health of any country is the great number of children and old persons which it contains. Lower Louisiana contains from eight to fourteen per cent more children in proportion to its population than Upper Louisiana, and from three to fourteen per cent. more old persons.

Again, for every one thousand white inhabitants between fifteen and fifty years of age, in all that part of Louisiana in which the floating plant is found, there are one hundred and thirty-two individuals between fifty and one hundred years of age—whereas, in all that part of Louisiana north of the region of the aquatic plant, the number of persons between fifty and one hundred years of age, is only one hundred and eighteen to the thousand adults.

In the famous city of Boston, the number of persons between fifty and hundred years of age is only one hundred and twenty to the thousand, twelve per cent. less than in the swamps and bayous of the most southern portion of Louisiana.

The city of New York contains only one hundred and ten to the thousand. But it is proper to observe that, according to the facts disclosed by the science of Political Arithmetic, all high and dry country situations, whether in the north or the south, remote from swamps, marshes and stagnant waters, are more favorable to human life between fifty and ninety years of age than low and damp situations; but after ninety years of age, the low and damp situations are the most friendly to human existence. Although the number of persons between fifty and ninety in Louisiana is less in proportion to the population than the number of persons between fifty and ninety in the northern States or in France, yet the number beyond ninety years of age in Louisiana is much greater than in any of the northern States or in France. Thus, according to the census of 1830, the city of New Orleans, with less than 70,000 inhabitants, contained more persons over one hundred years of age than New York and Philadelphia both together.

It is also a mistaken idea to suppose that dry and elevated situations in the south, remote from stagnant water, are less healthy, or less favorable to longevity than similar situations in the north. According to the facts afforded by Political Arithmetic, the lives of the people of a number of counties in Mississippi ought to be insured at a cheaper rate than the lives of the people of Kentucky, Ohio, Pennsylvania or New York. In Kentucky, for every one thousand inhabitants between fifteen and fifty years of age, there are one hundred and sixty-eight individuals between fifty and one hundred. In Ohio, one hundred and forty-eight. In Pennsylvania, one hundred and seventy-one. In New York State, one hundred and sixty-seven. In Lowndes county, Mississippi, among the white male inhabitants, for every thousand persons between fifteen and fifty, there are one hundred and ninety-one between fifty and one hundred; in Monroe county, Mississippi, one hundred and fifty-five; in Covington, one hundred and sixty-nine; in Lawrence, one hundred and seventy-six; in Pike, one hundred and sixty-eight; in Perry, one hundred and seventy-one; in Copiah, one hundred and sixty-five; in Amitie, one hundred and seventy-five; in Marion, two hundred; in Green, two hundred and twenty-five; in Jackson county, two hundred and thirty-two, and in Hancock, two hundred and forty-one.

The three last named counties contain more old people to the thousand than the State of Maine which contains two hundred and twenty one.

Hancock county exceeds Massachusetts by three in the thousand.

Although the probabilities of human life between fifty and ninety years of age are considerably less in the low swampy district of Louisiana, inhabited by the Jussieua Grandiflora, than in the dry and elevated counties of Mississippi, and less than in the high, dry and mountainous regions of Kentucky,

Pennsylvania and Vermont, yet it is fourteen in the thousand more favorable to longevity than similar low and marshy districts north of the region of the aquatic plant. It is even eighteen in the thousand more favorable to longevity than the low swampy State of Illinois.

But the hygienic properties of the Jussieua Grandiflora are less evident in promoting longevity, than in exempting man from that fatal tribe of malarious or miasmatic diseases which cut off so many of the southern people in the prime of life. The number of persons who die in the northern States, in the prime of life, by consumption, typhus fever and inflammatory complaints, is about equal, or even exceeds, the number who die in the south of malarious diseases. But the region of country, in which the Jussieua Grandiflora flourishes, is from its mild climate comparatively exempt from consumption, typhus and inflammatory affections; and by reason of the anti-malarious properties of the plant, it is singularly exempted from all that tribe of diseases which are produced by marsh miasmata or by the unwholesome air of swamps, called malaria. The plant, it would seem, converts into its own nutriment those very impurities of stagnant water, which in other situations load the atmosphere, at certain seasons of the year, with noxious effluvia.

I saw the identical plant, the Jussieua Grandiflora, which is so abundant in Lower Louisiana, cultivated as a curiosity in the botanical garden belonging to the University of Oxford. It had a large basin of water, several yards in diameter, appropriated exclusively to it. The water of the basin in which it floated was pure and sweet, while the water of a number of other basins, appropriated to other aquatic plants, was far from being as much so. The English botanist, who showed it to me, could not account satisfactorily for the circumstance.

He assured me that all the basins were filled at the same time. But the true explanation is, that the most of the other plants draw more or less nourishment from the earth and the atmosphere, and do not, as the Jussieua Grandiflora, derive the whole of their nourishment from the water. All aquatic plants, no doubt, serve to purify the water in which they grow in proportion to the amount of nourishment they draw from it.

I have already shown that the number of persons between thirty and forty years of age, in that part of Louisiana where the aquatic plant grows, is eight per cent. greater than the proportionate number of persons between thirty and forty in that section of Louisiana which lies north of the district inhabited by the plant—although the northern district includes a large portion of high and healthy piny woods.

Kentucky, in 1830, contained 87,852 white persons between twenty and thirty years of age, and only 49,750 between thirty and forty, and 31,442 between forty and fifty. The proportions, therefore, are as follows: For every hundred individuals in Kentucky, between twenty and thirty years of age, there are only fifty-six between thirty and forty. In the district of Louisiana inhabited by the aquatic plant, the proportional number is sixty-four: eight per cent. more persons die, therefore, in Kentucky, in the prime of life, than in the swamps of Lower Louisiana; but between forty and fifty, Kentucky has the advantage of Louisiana one per cent. The probabilities therefore of human life between thirty and fifty years of age, are seven per cent. better in the swamps of Lower Louisiana, than in the healthy State of Kentucky.

The State of Pennsylvania, in 1830, contained 237,257 white persons, between twenty and thirty years of age. If Pennsylvania were as favorable to human life, between thirty and forty years of age, as that part of Louisiana, in which the anti-malarious plant is indigenous, it ought to have contained 153,242 persons between thirty and forty. But the census shows that it contained only 144,776. Therefore 8466 persons, under forty, would have lived over forty, if they had resided in Lower Louisiana, instead of the mountains of Pennsylvania. But after forty, Pennsylvania has the advantage; but it is so small, as in twenty years, including the period from thirty to fifty, that the probabilities of human life are only one two hundred and thirty-seventh part greater in Pennsylvania than in Louisiana. But after fifty, the probabilities of the duration of life are considerably more in Pennsylvania until ninety, when it again turns decidedly in favor of Louisiana.

Ohio, in 1830, contained 156,864 inhabitants between twenty and thirty, and only 93,240 between thirty and forty; whereas, if Ohio were as favorable to human life, in its prime, as Lower Louisiana, it should have contained 101,424; making a difference in favor of the region of country inhabited by the Jussieua Grandiflora, of 8,184 persons. Even in twenty years, from thirty to fifty, the probability of human life would, in the same population, be 3379 better in Louisiana, than in Ohio. But after fifty, the probabilities would run in favor of Ohio until ninety, when it would turn in favor of Louisiana.

In regard to the health of children in Lower Louisiana, the following facts derived from the results afforded by political arithmetic, should be conclusive: For every thousand white females between fifteen and fifty in Lower Louisiana, there are 2357 children under fifteen years of age. If the country was not favorable to infantile existence, the number would not be so great, by one half.

In Pennsylvania, the number of children under fifteen years, to every thousand females, between fifteen and fifty, is only

2961. In Kentucky, 2370; in Ohio, 2275; in E. Tennessee, 1354; in the Northern District of New York, 1967; in Virginia, 1972; in Mississippi, 2540. But in the paludal, or swampy distrtcts of Mississippi, the number of children is about three hundred to the thousand less than in the district of Louisiana inhabited by the Jussieua Grandiflora.

In the United States, taken as a whole, the number of persons between twenty and thirty exceeds, in a small degree, the number of children under five years of age, (only sixteen in nearly two millions.) In Lower Louisiana, the children under five years, exceed the adults between twenty and thirty, forty-eight; in Upper Louisiana, the adults between twenty and thirty, exceed the children under five years, no less than 1208, giving Lower Louisiana about eight children, where Louisiana has seven. In the United States, the adults between thirty and fifty years of age, are nearly equal to the adults between twenty and thirty. The same thing occurs in that portion of Louisiana where the aquatic plant is found. There the adults between thirty and fifty are only forty-nine less than the adults between twenty and thirty. Whereas in Upper Louisiana, the adults between thirty and fifty fall short of the adults between twenty and thirty, no less than 1097.—Thus it appears, from a variety of facts, that a low, alluvial region, subject to annual inundation, and abounding in stagnant pools, lakes, lagoons and impassable swamps, in the most southern portion of the United States, is rendered not only habitable, but healthy, by an aquatic plant, that consumes or feeds upon the impurities of the water in which it grows, and thereby prevents the generation of miasmata, or those noxious vapors so unfriendly to human existence. It would seem, therefore, that the experiment of rendering other low and inundated districts of country healthy, by disseminating the Jussieua Grandiflora upon their stagnant waters, would be well worthy a trial. It would certainly be a great achievement of experimental philosophy, if she could extend the empire of her power into those low, swampy and insalubrious districts of the South, and convert them into healthy abodes; even to make mill-ponds healthy would be no small triumph.

True philosophy is not, as is too often supposed, an inactive, inanimate, inefficient thing, incapable of doing good, or of accomplishing useful purposes; but it is an animated, restless, self-moving power, perpetually in action, constantly striving to make inroads upon the mysteries of nature, in order to drag something to light which may subserve the purposes of utility to man. That proud Isle, on whose possessions the sun never goes down, and whose name is a terror to every land but our own, was formerly nothing but an unhealthy, insignificant spot of earth, noted for nothing but the crab-apple, and almost inaccessible by reason of stormy seas. Philosophy, however, by uniting itself, as an active and directive power, with the various artists, agriculturists and manufacturers, has made England what it now is. But there, as in all other countries, except our own, the happiness and comfort of the many have been sacrificed to the pride and luxury of the few. This, however, is not the fault of philosophy, but the fault of the people in not applying or calling in the aid of the experimental, or Baconian philosophy to direct the affairs of State.

Our happy form of government rests on the firm basis of the Baconian or experimental philosophy. Our individual distinctions in society are all such as nature makes. The broad, artificial distinctions which divide the people of Europe, are unknown amongst us. Our government is founded upon *natural*, not *artificial* distinctions in society. Our chief executive officer can never, in the nature of our philosophy, be a child in its cradle or a girl in her teens. The same experimental philosophy, which we have found to succeed so admirably in our government, will also succeed equally well in our fields; and cause two blades of grass to grow where only one grew before. It is not too much to hope from it, that it may yet be made to convert sickly districts into healthy ones. There are many ways of accomplishing this object, in a greater or less degree, by embankments, draining, and a proper system of cultivation. I only propose the dissemination of the Jussieua Grandiflora, as a means of preserving health in those districts not susceptible of being drained or cultivated. Mississippi already owes half her wealth and influence to the successful application of the experimental philosophy to a single plant. It has been but a few years ago, when the cultivators of the cotton plant considered one hundred pounds a good day's work, for any one laborer, and moreover thought themselves fortunate, if half the bowls or pods on the plant, did not annually perish with the disease called the rot.

At length the experimental philosophy, fortunately came to the aid of the planter. It procured some seed from another variety, or, as I believe, a different species of the cotton plant, as found in the table lands of Mexico. The pods of the Mexican species, were not affected by the rot, as the capsules were thicker, and less tender than the species at that time cultivated in Mississippi. But the attachment to the dissepiment and pillar of the capsule was so weak, that as soon as the capsules expanded, the cotton wool dropped out of the pods, and fell to the ground. The experiment having in a measure failed, a second was instituted, by planting the seed of both species together in the same drill. A new species of the cotton plant was thereby produced, which did not rot, did not drop out of the bowls or pods, yet the bowls expanded freely, making it more than twice as easy to pick or gather, as the kind formerly cultivated. But in a very few years it was found that the new made species of cotton, called Mexican, was fast degenerating into the old black seed kind. A third experiment was instituted, which consisted in separating the white, or Mexican seed from the black seed, and planting the white.—This was found to re-instate the new species which possessed the advantages of both the parent species without their disadvantages.

Thus, in a few years the experimental or Baconian philosophy, has enabled the planter to make twice the quantity of cotton with the same labor, and Mississippi is indebted to it for half her wealth and power.

Our government, as I said, is founded upon the same philosophy—our agriculture is daily improving under its influence—by its aid our manufacturing establishments are already beginning to rival those of Europe—it has increased the velocity, beauty and strength of our shipping beyond all other nations in the world. It has diffused science into mechanism, shortened the road to mechanical knowledge, and elevated the American mechanic to the high station of the intelligent, the good, and the useful. It has discovered better systems of education, improved those that formerly existed, and established new ones. Sunday Schools, Bible and Tract Societies and Missionary establishments are nothing more than so many philosophical experiments, having for their object the diffusion of Christianity—or in other words, experimental philosophy trying to open the adamantine doors of ignorance and superstition to let in light. The ecclesiastical page of future times will tell the result. If this experimental philosophy, which has already done so much for mankind, and which is daily doing more, be invoked, it will not be expecting too much from it to hope, that it will ultimately point out the means, by which unhealthy districts of country may be rendered salubrious. Whether the dissemination of the Jussieua Grandiflora be one of them, time and experience can alone determine.

